# SARS-CoV-2 Polymerase Chain Reaction Cycle Threshold Trends in Patients Who Are Immunocompromised and Implications for Isolation Precautions

**DOI:** 10.1093/ofid/ofae367

**Published:** 2024-06-29

**Authors:** Courtney E Harris, Vineeta Vaidya, Zhou Lan, Michael Klompas, Chanu Rhee, Lindsey R Baden, Meghan A Baker

**Affiliations:** Division of Infectious Disease, Medical University of South Carolina, Charleston, South Carolina, USA; Infection Control Department, Brigham and Women's Hospital, Harvard Medical School, Boston, Massachusetts, USA; Center for Clinical Investigation, Brigham and Women's Hospital, Harvard Medical School, Boston, Massachusetts, USA; Division of Infectious Diseases, Brigham and Women's Hospital, Harvard Medical School, Boston, Massachusetts, USA; Division of Infectious Diseases, Brigham and Women's Hospital, Harvard Medical School, Boston, Massachusetts, USA; Division of Infectious Diseases, Brigham and Women's Hospital, Harvard Medical School, Boston, Massachusetts, USA; Division of Infectious Diseases, Brigham and Women's Hospital, Harvard Medical School, Boston, Massachusetts, USA

**Keywords:** COVID-19, cycle threshold, immunocompromised, infection control, SARS-CoV-2

## Abstract

Among 495 patients who were immunocompromised and tested positive for SARS-CoV-2, polymerase chain reaction cycle thresholds remained <33 beyond 20 days more frequently in patients with hematologic malignancies, particularly those receiving B-cell–depleting or Bruton tyrosine kinase inhibitor therapy, as compared with those with solid organ malignancy (26% vs 5%).

## Teaser

Among 495 immunocompromised patients positive for SARS-CoV-2, PCR cycle thresholds remained <33 beyond 20 days more frequently in patients with hematologic malignancies, particularly those receiving B-cell depleting or BTKi therapy, compared to those with solid organ malignancy (26% vs 5%).

## Graphical Abstract

**Graphical Abstract:**
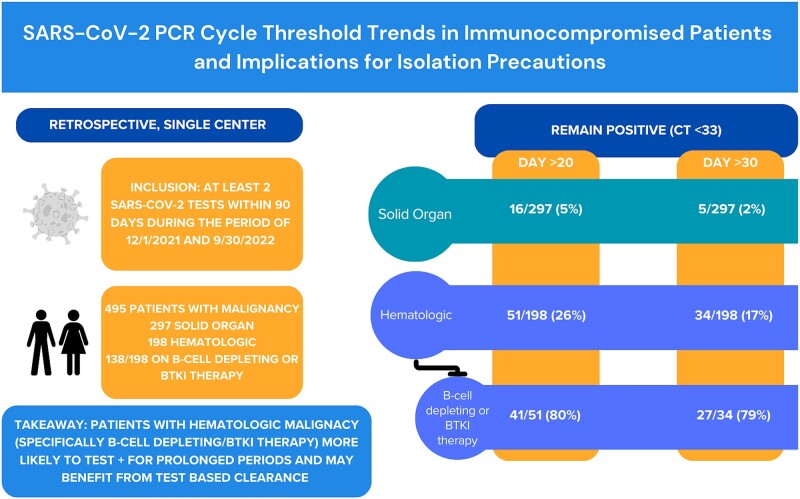

The Centers for Disease Control and Prevention and other public health agencies recommend different periods of isolation for patients with immunocompromised vs nonimmunocompromised status [1]. However, most of these policies do not delineate differences in isolation based on the different categories of immunocompromised populations.

Real-time reverse transcriptase polymerase chain reaction (PCR) is a primary method to test for SARS-CoV-2 due to its high sensitivity and specificity, amplifying the detected virus in a quantitative manner, with the cycle threshold (Ct) value being the number of cycles that are required to detect viral RNA. Ct values are inversely proportional to the viral load, with Ct values >33 to 35 correlating with negative viral culture results and thus being a credible proxy for potential infectivity [2–4]. No differences in Ct values based on demographic characteristics such as sex, race, or ethnicity have been found [5], but age, comorbidities, solid organ transplantation, and immunocompromised status have been associated with delayed clearance [6]. However, there are limited data on differences in clearance among patients who are immunocompromised. Furthermore, vaccination may affect the Ct value trajectory, with prior literature showing that those infected after vaccination had a shorter mean time to clearance as compared with those not vaccinated [7, 8].

Our study aimed to assist clinicians in understanding the duration of infectivity based on the underlying immunocompromised status of the patient to inform testing strategies and infection prevention measures to create clinic and hospital protocols and better inform public health policies for the future based on this specialized patient population.

## METHODS

We performed a single-center retrospective study of all inpatients and outpatients with solid or hematologic malignancies at the Dana-Farber Cancer Institute (DFCI) and Brigham and Women's Hospital between 1 December 2021 and 30 September 2022 (Omicron-predominant period). This study was approved by the Brigham and Women's Hospital and DFCI institutional review boards.

Patients with at least 1 outpatient visit to DFCI during the study period and a positive PCR test result for SARS-CoV-2 with a value ≤30 were included if they had at least 2 tests on separate days within 90 days of each other and with Ct values reported. The practice at both hospitals was to serially test patients in the inpatient and outpatient settings to clear precautions. Two tests with a Ct value >33 or passage of at least 20 days were required to allow discontinuation of precautions. All tests were included during the study period, and if 2 tests were performed in 1 calendar day, the test with the lower Ct value was chosen. All testing was run on the Cepheid or Panther platform per the manufacturer’s specifications. In many cases, the same test system was used for follow-up testing, but due to constraints or testing location, this was not always the case. We characterized the possible duration of infectivity using Ct values ≤33 as proxies for potential infectivity. Tests were included until there were 2 consecutive results with Ct values >33, at which point the patient was no longer considered infectious with SARS-CoV2. If multiple tests occurred during the time frame, the result with the lowest value was used.

Data were extracted from the medical record to determine if patients had a condition on their problem lists or had received a medication in the past 6 months that classified them as being immunocompromised (Supplementary Tables 1 and 2). These medications included B-cell–depleting therapy or Bruton tyrosine kinase inhibitors (BTKis), medications associated with persistent infection. Patients were excluded if they did not meet these criteria, met only the high-risk medication criteria, or did not have an underlying malignancy on further chart review.

Patient information was analyzed as follows: demographics, testing, reason for compromised immunity (solid organ malignancy vs hematologic malignancy), B-cell–depleting therapy and BTKi treatment in the 6 months prior to SARS-CoV-2 infection, and vaccination status. Between-group differences were calculated by analysis of variance and were based on the duration of SARS-CoV-2 positivity by the number of vaccinations and the time from the last vaccination to the first positive test result. The normality assumption of residual in the analysis of variance model is validated by the Shapiro normality test. The cubic polynomial regression with a tricube function for weighting was used to draw the smooth curve over time points. The significance level is 5% and 2-sided, and R software (version 4.2.0) was used.

## RESULTS

Among 494 patients with 495 episodes of SARS-CoV-2 infection, 297 (60%) had solid malignancies, and 198 (40%) had hematologic malignancies, of whom 135 (68%) had received B-cell–depleting or BTKi therapy. Supplementary Appendix Table 1 shows patients’ demographics. The volume of testing declined over time since infection largely as patients turned to negative status, and by day 20, 289 (59%) had either a negative test result or a Ct value ≤33 (Supplementary Appendix Table 2).

**Figure 1.**
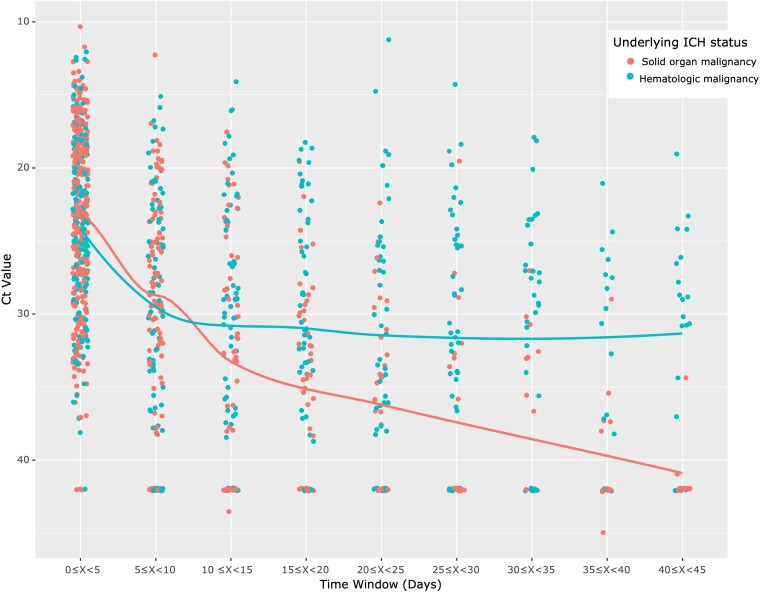
SARS-CoV-2 cycle threshold over time by ICH status. Each 5-day period includes all tests available, and each point in the time window represents 1 patient. Tests from patients with solid organ malignancies and tests from patients with hematologic malignancies are shown. Negative values were assigned a value of 42. If a patient had more than 1 test within a window, the lowest value was chosen. Ct, cycle threshold; ICH, immunocompromised host.

Table 1 displays patient-level data demonstrating the number of positive test results by Ct value in 5-day intervals stratified by immunocompromising conditions. After day 10, 41 of 297 (14%) patients with solid organ malignancy tested positive vs 76 of 198 (38%) with hematologic malignancies. Among patients with hematologic malignancies and on B-cell–depleting or BTKi treatment, 58 (43%) continued to test positive after day 10. After day 20, only 16 (5%) patients with solid organ malignancy tested positive. However, 51 (26%) patients with hematologic malignancies tested positive after day 20, and among those undergoing B-cell–depleting or BTKi treatment, 41 (30%) tested positive. Figure 1 presents a comparison of trends in Ct value based on tests in the time windows.

**Table 1.** SARS-CoV-2 PCR Testing Ct Positivity Over Time

	Day >5	Day >10	Day >15	Day >20	Day >25	Day >30	Day >35	Day >40
Ct value: <20^a^	38	38	16	12	7	3	2	2
Solid	14	14	1	1	1	0	0	0
Hematologic (CD20/BTKi)^a^	24 (20)	24 (20)	15 (12)	11 (10)	6 (5)	3 (2)	2 (1)	2 (1)
Ct value: 20 to <25^a^	52	28	19	15	14	13	9	7
Solid	22	7	3	1	0	0	0	0
Hematologic (CD20/BTKi)^a^	30 (23)	21 (19)	16 (15)	14 (12)	14 (13)	13 (12)	9 (8)	7 (7)
Ct value: 25 to <30^a^	59	42	33	23	20	16	12	9
Solid	34	18	15	8	4	3	2	1
Hematologic (CD20/BTKi)^a^	25 (17)	24 (17)	18 (13)	15 (10)	16 (11)	13 (9)	10 (8)	8 (6)
Ct value ≥30^a^	85	71	69	59	41	32	27	26
Solid	39	38	32	29	22	20	15	14
Hematologic (CD20/BTKi)^a^	46 (32)	33 (22)	37 (24)	30 (23)	19 (16)	12 (9)	12 (11)	12 (11)
Ct value <33^b^								
Total	181 (37)	117 (24)	91 (18)	67 (14)	54 (11)	39 (8)	31 (6)	27 (5)
Solid	85 (29)	41 (14)	26 (8)	16 (5)	8 (3)	5 (2)	3 (1)	2 (1)
Hematologic	96 (48)	76 (38)	65 (33)	51 (26)	46 (23)	34 (17)	28 (14)	25 (13)
CD20/BTKi	72 (53)	58 (43)	50 (37)	41 (30)	37 (27)	27 (20)	23 (17)	21 (16)

If multiple Ct values were present after day *x*, the lowest value was used.

Abbreviations: BTKi, Bruton tyrosine kinase inhibitor; Ct, cycle threshold; PCR, polymerase chain reaction.

^a^Data are presented as No. Data in parentheses indicate the number of patients undergoing CD20/BTKi therapy.

^b^Data are presented as No. (%). Percentages are based on the total number of patients in the disease class (eg, solid organ malignancy has a denominator of 297).

The majority of patients (55%) had ≥3 vaccines at least 1 week prior to infection and were considered fully vaccinated by Centers for Disease Control and Prevention guidance for hosts who are moderately to severely immunocompromised. Vaccination data were analyzed by comparing group differences in the mean duration of RNA shedding by the number of vaccinations and by the mean timing of vaccination prior to diagnosis of SARS-CoV-2 infection (Supplementary Appendix Table 3), with medians reported. The median duration of viral RNA shedding decreased with increasing vaccine doses (*P* = .01).

## DISCUSSION

On retrospective review of SARS-CoV-2 reverse transcriptase PCR Ct values in a large sample of patients with underlying malignancy, we found that almost all patients with solid malignancies had cleared the virus 20 days after their first positive test result, whereas almost a third of patients with hematologic malignancies had not. Our study suggests that public health officials should consider differentiating clearance guidance for patients with hematologic vs solid malignancies. Given the high fraction of people with hematologic malignancies who had persistently low Ct values >20 days after their first positive result, test-based clearance should be favored for this population, whereas time-based clearance based on a window >20 days may be reasonable for patients with solid malignancies.

Retrospective studies in other patients who are immunocompromised have suggested that test-based strategies may be warranted in patients with solid organ transplantation [6]. Furthermore, Li et al [9] determined that the median nasal viral and culture clearance in a group with severe hematologic malignancy was 72 days, significantly longer than in nonsevere and nonimmunocompromised groups. The authors found that the severe group had diminished SARS-CoV-2–specific humoral immunity as well as reduced T-cell–mediated responses, suggesting that deficient B- and T-cell responses are associated with the risk of persistent infection. Although our study did not follow patients beyond day 40, our results are consistent with those of Li et al: the hematologic malignancy group described in this study was at the highest risk of persistent infection. Finally, this study highlights that the number of vaccinations also affects the median duration of RNA shedding in patients who are immunocompromised.

Our study provides evidence that a time-based strategy alone may be sufficient for patients with solid malignancies. A better understanding of the trend of Ct values in patients who are immunocompromised may be used to help guide the timing of further chemotherapy or hematopoietic stem cell transplantation.

Possible limitations of our study include the use of Ct values as a proxy for potential infectivity, variability in test results by specimen quality, sample site, and assay performance [10, 11]. The same sample run on different assays may have a difference in several Ct value points between assays, which may affect Ct cutoff values [12]. While the laboratory did compare differences between platforms and determine that the tests could be used interchangeably, we recognize that there are subtle differences and we did not have complete data on the platform used for each test. While variability in PCR-based testing is recognized, there are no alternatives with similar sensitivity, widespread availability, and manageable costs. Antigen testing is less sensitive than PCR and correlates variably with viral culture. Viral culture is rarely available, expensive, and slow to result. We are also limited by differences in testing frequency and cadence among patients, with some patients tested more frequently, particularly in the inpatient setting. However, this simulates real-world experiences and how our clinicians will use these tests going forward. Similarly, we did not control for age, comorbidities, or other factors that could affect clearance, as our goal was to see if immunocompromised host status alone could help infection control policies. Different forms of “active treatment” for solid organ malignancy, as well as treatments for hematologic malignancy other than BTKi or B-cell–depleting treatment, were not analyzed separately, as the sample sizes of subgroups may be limiting and were more difficult to classify consistently. We did not obtain data on symptomatic improvement with disease severity, which could enhance the strength of these insights in future studies, and testing performed outside of our system, as well as vaccinations that were not recorded in the medical record, was not available for analysis.

## CONCLUSION

Patients with solid organ malignancy were unlikely to have a positive result upon SARS-CoV-2 reverse transcriptase PCR Ct value testing after 20 days from the first positive test result. Thus, time-based strategies for discontinuing precautions may be a valid approach for this population. However, almost one-third of patients with hematologic malignancies, particularly those with B-cell–depleting or BTKi therapy, tested positive with Ct values in the potentially infectious range >20 days after their first positive test results, suggesting that test-based clearance may be preferable for this population.

## Supplementary Material

ofae367_Supplementary_Data

## Supplementary Data

Supplementary materials are available at *Open Forum Infectious Diseases* online. Consisting of data provided by the authors to benefit the reader, the posted materials are not copyedited and are the sole responsibility of the authors, so questions or comments should be addressed to the corresponding author.

## Notes

***Financial support.*** This work was supported in part by the Chleck Family Foundation.
