# Supplementary material for: SARS-CoV-2 Polymerase Chain Reaction Cycle Threshold Trends in Patients Who Are Immunocompromised and Implications for Isolation Precautions
Source: Open Forum Infect Dis. 2024 Jun 29;11(7):ofae367. doi: 10.1093/ofid/ofae367 (PMC11285146; doi:10.1093/ofid/ofae367)
Supplement: ofae367_Supplementary_Data [file ofae367_supplementary_data.zip › Appendix Ct Value.docx]

**Appendix**

Table 1 Patient Demographics

|  | Solid organ malignancy  (n=297) | Hematologic malignancy  (n=198) | Total  (n=495) |
| --- | --- | --- | --- |
| **Age Mean, SD** | 63.6 (14.3) | 61.8 (15) | 62.9 (14.6) |
| **Age Median, IQR** | 65.1 (20.4) | 65.2 (17.5) | 65.1 (18.8) |
| **Female** | 169 (57%) | 88 (44%) | 257 (52%) |
| **Race** |  |  |  |
| Asian | 19 (6%) | 5 (3%) | 24 (5%) |
| Black/African American | 33 (11%) | 14 (7%) | 47 (9%) |
| White | 221 (74%) | 163 (82%) | 384 (78%) |
| Other | 20 (7%) | 14 (7%) | 34 (7%) |
| Missing | 4 (1%) | 2 (1%) | 6 (1%) |
| **Hispanic/Latino** | 20 (7%) | 16 (8%) | 36 (7%) |

Table 2 Overview of SARS-CoV-2 PCR testing

| Time Window (Days) | Number of Patients with Any Future Follow up Test | Number of Patients with Test in Time Window | Total number of Tests in Time Window | Number of Positive Tests  (Ct<33) | Mean (SD) Ct value^*^  (Ct <33) | Number of Patients Who Have at Least one Negative Test or test with a Ct=>33 (Cumulative) |
| --- | --- | --- | --- | --- | --- | --- |
| 0 ≤ X < 5 | 495 | 495 | 813 | 635 | 23.4 (5.2) | 94 |
| 5 ≤ X < 10 | 427 | 180 | 245 | 164 | 25.5 (4.3) | 162 |
| 10 ≤ X< 15 | 359 | 128 | 166 | 87 | 25.8 (4.7) | 226 |
| 15 ≤ X< 20 | 312 | 100 | 139 | 65 | 27.0 (4.5) | 289 |
| 20 ≤ X< 25 | 276 | 81 | 122 | 45 | 26.2 (4.6) | 351 |
| 25 ≤ X< 30 | 245 | 71 | 98 | 41 | 26.9 (4.7) | 399 |
| 30 ≤ X< 35 | 206 | 47 | 67 | 32 | 27.1 (4) | 426 |
| 35 ≤ X< 40 | 185 | 33 | 41 | 13 | 27.7 (3.1) | 452 |
| 40 ≤ X< 45 | 173 | 34 | 39 | 16 | 27.8 (3.6) | 475 |

^*^Negative tests were not given a value, mean values are for measurable virus/positive values only

Table 3 Vaccination Status and SARS-CoV-2 Duration of RNA Shedding

|  | | **n** | **Mean**  **(SD)** | **Median**  **[Min, Max]** | **p-value^#^** |
| --- | --- | --- | --- | --- | --- |
| **Duration of SARS-CoV-2 RNA shedding (days) by number of vaccinations** | **0 vaccines** | 88 | 59 (77.7) | 29.5 [0.4, 488] | 0.01 |
|  | **1 vaccine** | 38 | 42.2 (43.5) | 26.7 [0.9, 192] |  |
|  | **2 vaccines** | 99 | 34 (38.8) | 24.8 [0.3, 260] |  |
|  | **3 vaccines** | 233 | 41.4 (47.3) | 23 [0.02, 232] |  |
|  | **≥4 vaccines** | 40 | 31.6 (38.5) | 16.8 [1.1, 215] |  |
| **Duration of SARS-CoV-2 RNA shedding by time from last vaccine dose to SARS-CoV-2 first positive test^*^** | **0-3 months** | 107 | 40.2 (47.4) | 24.9 [0.02, 232] | 0.044 |
|  | **3-6 months** | 127 | 47.5 (53.5) | 26.5 [0.6, 260] |  |
|  | **6-9 months** | 85 | 31.8 (30.7 | 22.1 [0.3, 171] |  |
|  | **9-12 months** | 58 | 30.9 (37.2) | 14.5 [0.9, 156] |  |
|  | **>12 months** | 25 | 30.6 (29.4) | 21 [0.7, 104] |  |

*Duration of SARS-CoV-2 RNA shedding is defined as the duration between the first and last test result (or until the first negative)

^#^P-value of mean values
