# Supplementary material for: SARS-CoV-2 Polymerase Chain Reaction Cycle Threshold Trends in Patients Who Are Immunocompromised and Implications for Isolation Precautions
Source: Open Forum Infect Dis. 2024 Jun 29;11(7):ofae367. doi: 10.1093/ofid/ofae367 (PMC11285146; doi:10.1093/ofid/ofae367)
Supplement: ofae367_Supplementary_Data [file ofae367_supplementary_data.zip › Supplementary Data.docx]

**Supplementary Data**

Table 1 List of Immunocompromised Conditions Extracted from EMR problem list

| - Hematopoietic stem cell transplant |
| --- |
| - - Stem cell transplant |
| - - Allogenic |
| - - Autologous |
| - - Cord blood |
| - CAR-T-cell therapy (chimeric T-cell therapy) |
| - Graft versus host disease (GVHD) |
| - Solid organ transplant (SOT) |
| - - Heart transplant |
| - - Lung transplant |
| - - Renal transplant |
| - - Liver transplant |
| - - Small bowel transplant |
| - - Pancreas transplant |
| - Hematologic malignancy |
| - - Leukemia |
| - - - Acute myeloid leukemia |
| - - - Acute lymphocytic leukemia |
| - - Lymphoma |
| - - - Non-Hodgkin lymphoma |
| - - - Hodgkin lymphoma |
| - - Multiple myeloma |
| - - Myeloproliferative disorder |
| - - Erdheim-Chester disease |
| - Any solid malignancy (cancer, carcinoma) |
| - - Brain cancer |
| - - - Glioblastoma |
| - - - Astrocytoma |
| - - - Brainstem glioma |
| - - - Ependymoma |
| - - - Glioma |
| - - - Medulloblastoma |
| - - - Neuroblastoma |
| - - - Oligodendroglioma |
| - - Head and neck cancer |
| - - - Lip cancer |
| - - - Oral cancer |
| - - - Throat cancer |
| - - - Nasopharyngeal carcinoma |
| - - - Nasal cavity ccancer |
| - - - Pharyngeal cancer |
| - - - Salivary gland cancer |
| - - - Hypopharyngeal cancer |
| - - - Esophageal cancer |
| - - - Oropharyngeal cancer |
| - - - Tongue cancer |
| - - Gastrointestinal cancer |
| - - - Appendix cancer |
| - - - Cholangiocarcinoma |
| - - - Gallbladder cancer |
| - - - Stomach cancer |
| - - - - Gastric cancer |
| - - - - Gastrointestinal stromal tumor |
| - - - - Gastrointestinal stromal tumor |
| - - - Liver cancer |
| - - - - Hepatocellular carcinoma |
| - - - Pancreatic cancer |
| - - - - Islet cell cancer |
| - - - Colorectal cancer |
| - - - - Colon cancer |
| - - - - Rectal cancer |
| - - - Anal cancer |
| - - Breast cancer |
| - - - Invasive lobular carcinoma |
| - - - Tubular carcinoma |
| - - - Medullary carcinoma |
| - - - Mammary carcinoma |
| - - Melanoma |
| - - Non-melanoma skin cancer |
| - - - Squamous cell carcinoma |
| - - - Basal cell carcinoma |
| - - Mesothelioma |
| - - Lung cancer |
| - - - Non-small cell lung cancer |
| - - - Small cell lung cancer |
| - - - Adenocarcinoma |
| - - Kidney (renal) cancer |
| - - Sarcoma |
| - - Kaposi sarcoma |
| - - Eye |
| - - - Retinoblastoma |
| - - - Optic nerve glioma |
| - - Endocrine cancer |
| - - - Thyroid cancer |
| - - - Adrenocortical carcinoma |
| - - - Pheochromocytoma |
| - - - Merkel cell carcinoma |
| - - Genitourinary cancer & gynecologic cancer |
| - - - Bladder cancer |
| - - - Prostate cancer |
| - - - Cervical cancer |
| - - - Vaginal cancer |
| - - - Endometrial cancer |
| - - - Ovarian cancer |
| - - - Penile cancer |
| - - - Kidney cancer |
| - - - Renal cell carcinoma |
| - - - Urethral cancer |
| - - - Uterine cancer |
| - - - Vulvar cancer |
| - - - Wilms tumor |
| - - - Nephroblastoma |
| - - - Transitional cell carcinoma |
| - - - Testicular cancer |
| - - - Germ cell tumor |

Table 2 Medications used in last 6 months

| - Rituximab |
| --- |
| - Ocrelizumab |
| - Ofatumumab |
| - Alemtuzamab |
| - Daratumamab |
| - Obintuzumab |
| - Isatuximab |
| - Ubilutximab |
| - Ibrutinib |
| - Acalabrutinib |
| - Zanubrutinib |
| - Pirtobrutinib |
